# Enhancing the Mn-Removal Efficiency of Acid-Mine Bacterial Consortium: Performance Optimization and Mechanism Study

**DOI:** 10.3390/microorganisms11092185

**Published:** 2023-08-30

**Authors:** Dongmei Hou, Lan Zhang, Chuncheng Li, Lutong Chen, Jianping Zou

**Affiliations:** National-Local Joint Engineering Research Center of Heavy Metals Pollutants Control and Resource Utilization, Nanchang Hangkong University, Nanchang 330063, China; zl15103424308@163.com (L.Z.); 15847761908@163.com (C.L.); 1780679929@126.com (L.C.)

**Keywords:** bioremediation, network analysis, response surface methodology, bacterial consortium, biofilter

## Abstract

In this study, an acclimated manganese-oxidizing bacteria (MnOB) consortium, QBS-1, was enriched in an acid mine area; then, it was used to eliminate Mn(Ⅱ) in different types of wastewater. QBS-1 presented excellent Mn removal performance between pH 4.0 and 8.0, and the best Mn-removal efficiency was up to 99.86% after response surface methodology optimization. Unlike other MnOB consortia, the core bacteria of QBS-1 were *Stenotrophomonas* and *Achromobacter*, which might play vital roles in Mn removal. Besides that, adsorption, co-precipitation and electrostatic binding by biological manganese oxides could further promote Mn elimination. Finally, the performance of the Mn biofilter demonstrated that QBS-1 was an excellent inoculant, which indicates good potential for removing Mn contamination steadily and efficiently.

## 1. Introduction

Manganese (Mn) is an essential trace element for human health, but excessive intake of it will induce chronic poisoning in humans, eventually causing serious damage to the liver, lungs, and other organs [1,2,3]. The World Health Organization’s (WHO) recommended amount of Mn in drinking water is 0.4 mg/L [4]. And in China, the concentration of Mn should not exceed 0.1 mg/L, which was stipulated by the national sanitary standard for drinking water [5,6]. Therefore, the prevention and control of manganese pollution in water bodies has always been the research focus of environmental workers.

Traditional methods for Mn removal are mainly focused on physicochemical approaches, such as sorption, precipitation, membrane filtration, oxidation and so on [7,8]. Although these methods had been proved to be effective, there were still some problems that needed to be overcome, such as complicated processes, secondary pollutants, high cost and low economic benefit [9]. As an alternative, microbial manganese oxidation has drawn much attention in recent years, since it has the advantages of simpler, low-cost and higher efficiency [10,11,12]. Mn-oxidizing bacteria (MnOB) can catalyze Mn(II) oxidation and precipitate as biogenic Mn oxides, which have been successfully used in purifying groundwater and industrial wastewater [13,14,15]. According to reports, manganese was effectively eliminated by MnOB when the concentration did not exceed 100 mg/L. However, if the concentration exceeds this limit, the removal efficiencies will decrease noticeably. This is particularly true for increasing Mn concentrations, which may have toxic effects on microorganisms [16]. Thus, the limitation of previous studies was that the most isolated bacteria could not tolerate the higher concentrations of Mn in real wastewater. More importantly, the reported MnOB used in the remediation were mostly pure cultured bacteria, in which the removal efficiency was not stable and the bacterial community system was easily destroyed by the changing environment [17,18,19]. Since the above-mentioned disadvantages have greatly limited the applications of these bacteria in environmental remediation, additional MnOB with high tolerance and stability urgently need to be discovered.

The extreme conditions of mine areas provide an excellent breeding ground for resistant bacteria, which endow the indigenous bacteria with several special mechanisms for high heavy metal and low pH stress [20,21]. To obtain the high-tolerance bacteria, some MnOB were isolated from a mine environment, such as *Duganella* sp., *Albidiferax* sp., *Bacillus* sp. and *Stenotrophomonas* sp., and *Lysinibacillus* sp. [22,23,24]. Nevertheless, limited work has focused on the function and possible application of mine Mn(II)-oxidizing consortium. Moreover, the metabolic and removal mechanisms of these bacteria are still unclear and need further investigation [25].

Hence, the aims of this study are as follows: (1) obtain a Mn(II)-oxidizing bacterial consortium from a highly polluted mine area, and then improve the manganese-oxidizing efficiency by response surface methodology (RSM); (2) establish the key bacteria and elucidate the metabolic and removal mechanisms; (3) treat with different kinds of Mn-contaminated water to examine the efficiency and stability of this bacterial consortium. These findings may allow for a better understanding of the microbial communities that influence the biogeochemical cycling of Mn and provide a potential strategy for treating manganese-laden wastewaters.

## 2. Materials and Methods

### 2.1. Acclimation of Mn(II)-Oxidizing Consortium

The MnOB consortium was screened from the sediment in Qibaoshan mine, Hunan province, China (113°55′16″ E, 28°16′54″ N). The physicochemical properties of the sediment sample were pH 4.71, Mn 1.07 × 10^3^ mg/Kg, Fe 7.56 × 10^4^ mg/Kg, Cu 1.5 × 10^3^ mg/Kg, Zn 1.9 × 10^3^ mg/Kg and Cd 8.99 mg/Kg. Slurry (5 g) was added to 100 mL of autoclaved PYCM medium with 0.1 g/L MnSO_4_·H_2_O [26]. The mixture was incubated for 14 days at 170 rpm, at 30 °C. Then, the bacterial suspension (10%, *v*:*v*) turned into a fresh medium. About 1 month later, the enrichment culture was obtained when the bacteria density reached 1 × 10^9^ cells/mL and the leucoberbelin-blue (LBB) colorimetric assay was blue [27]. After enrichment, the MnOB consortium was acclimated by serial sub-culturing to have a good Mn resistance. The details are shown in Supplementary Materials.

### 2.2. Optimization of the Culture Conditions

The 2 mL active bacterial suspension (10^9^ cells/mL) was inoculated into 100 mL of PYCM medium. The control group was run under the same conditions but with no bacteria added. The effects of pH (3.0, 4.0, 5.0, 6.0, 7.0, 8.0 and 9.0), temperature (10, 15, 20, 25, 30, 35 and 40 °C), inoculum size (0.5, 1.0, 2.0, 3.0, 4.0, 5.0%), rotary speed (80, 110, 140, 170, 200 rpm), initial concentration of Mn(II) (100, 200, 400, 500, 600, 700 and 800 mg/L) on the biomass of bacterial consortium and Mn-removal efficiency were investigated. Moreover, the effect of common coexisting metal ions (Fe^3+^, Cu^2+^, Zn^2+^, Cd^2+^) in wastewater on Mn removal were investigated by adding FeCl_3_, CuCl_2_, ZnSO_4_ and CdCl_2_ to standard PYCM medium, respectively. The final concentrations of coexisting metal ions were set as 1, 10, 100 mg/L. And all of the groups were operated at 30 °C, pH = 7.0 and 170 rpm for 14 days.

After that, three major independent variables (temperature, pH and inoculum size) were tested in an 18-run experiment with a Box–Behnken design (BBD) (Tables S1 and S2). The experimental design and data analysis were carried out by the Design-Expert Version 8.0.6 software [28]. More details are shown in Supplementary Materials.

### 2.3. Batch Experiments

The batch experiments were carried out in a 2-week period with the consortium QBS-1 and the culture conditions as follows: work volume, 150 mL; inoculum size, 4% (*v*/*v*); temperature, 30 °C; pH, 7.0; rotary speed, 170 rpm; final concentration of Mn(Ⅱ), 600 mg/L. The control group was established without bacteria, and all the experiments were carried out with three replications. Aliquots were collected every two days for cell density, pH and manganese concentration measurements. The biogenic Mn oxides (BMO) were harvested on the 2nd day and 14th day by centrifuging 50 mL suspension (8000× *g*, 10 min) and washed with double-distilled water before vacuum freeze-drying, and then stored in a dryer before analysis.

### 2.4. Microbial Community Analysis

For microbial community analysis (2nd day, 7th day and 14th day), the 16S rRNA V3-V4 gene was selected as amplified fragment, and the primers were as follows: 314F (5′-CCTAYGGGRBGCASCAG-3′) and 806R (5′-GGACTACNNGG GTATCTAAT-3′) [29,30]. After amplification, 16S rRNA gene segments were sequenced on an Illumina HiSeq platform. For details of analysis methods, refer to Supplementary Materials and our previous studies [26]. The raw sequences were all deposited in the NCBI database, and the accession ID was SUB7658358.

### 2.5. Column Experiment

**Figure 1:**
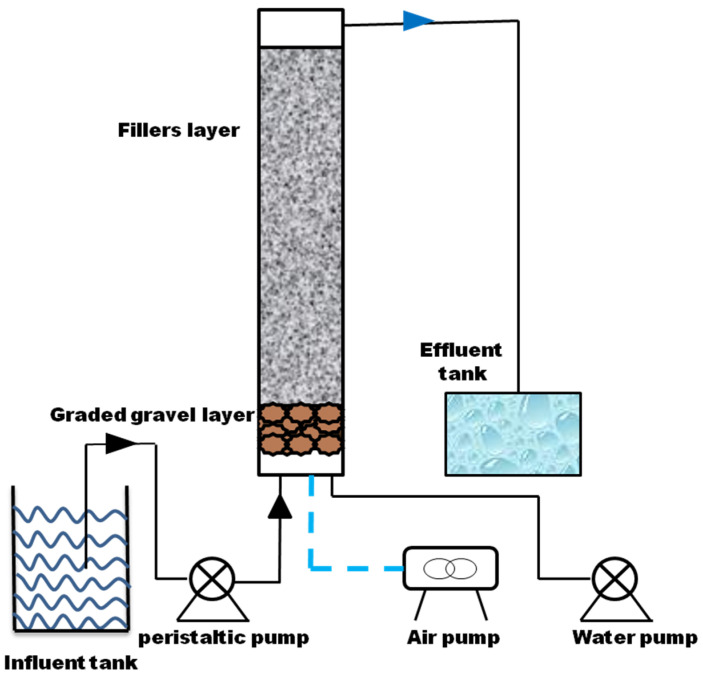
Schematic diagram of the biofilter reactor. (Column characteristics: active height: 50 cm, diameter: 12 cm, filler layer: 25 cm, graded gravel layer (pebble): 8 cm).

A lab-scale biofilter was established to examine the continuous effect of the consortium on the real wastewater. Three different kinds of wastewater were examined in this part of the experiment (Table S3). The filter consisted of Plexiglas tube, peristaltic pump, water pump, influent and effluent tank (Figure 1). A filler layer was added with ceramic particles, and the graded gravel layer was pebble. Then, 500 mL of active culture were applied as inoculum. Synthetic Mn-contaminated wastewater (Table S4) was added to fill the reactor as nutrient supply. The start-up of biofilter consisted of two steps: firstly, the column was seeded with synthetic Mn-contaminated wastewater at a low flow rate (0.15 L h^−1^) to enrich microorganism; 15 days later, the reactor influent was replaced by real wastewater, and the flow rate increased to 0.21 L h^−1^, hydraulic retention time (HRT) was 24 h. The backwashing time was 50 days. During the start-up period, the concentrations of Mn in the influent and effluent were analyzed every day.

### 2.6. Analytical Methods

The cell densities of bacteria were analyzed by a UV-visible spectrometer at 660 nm. The pH of culture was measured immediately using a pH meter. The final concentration of heavy metal ions was measured using inductively coupled plasma mass spectrometry (ICP-MS) [31]. The Mn(II) oxidation capacity was determined through the leucoberbelin method (LBB) [32] and the removal efficiency of heavy metals (Q_e_) was estimated by formulas as follows.

(1) $$Qe=\frac{C_{0}-C_{1}}{C_{0}}\times 100\%$$

where C_0_ was the concentration of heavy metals before culturation and C_1_ was the concentration of heavy metals after culturation.

The morphological changes of BMO and bacteria were analyzed by scanning electron microscope coupled with dispersive X-ray analysis (SEM-EDX) (Hitachi SU-8020, Tokyo, Japan) and transmission electron microscopy (TEM, G2F20, Hillsboro, OR, USA). The specific surface area (SSA) of BMO was measured using N_2_ adsorption (ASAP-2020, Atlanta, GE, USA) and calculated from the Brunauer–Emmett–Teller (BET) equation. For X-ray diffraction (XRD, Ultimate 4, Rigaku, Akishima, Japan) analysis, Mn oxides were scanned over a range of 2θ from 5° to 80° equipped with CuKα radiation. X-ray photoelectron spectroscopy (XPS) analysis of BMO was achieved using a Mg Ka X-ray source (1253 eV) and a base pressure of 3 × 10^−9^ Torr in the analytical chamber (Ulvac-Phi, Chigasaki, Japan). FTIR spectroscopy analysis of BMOs was performed by a Vertex 70v spectrometer (Bruker, Mannheim, Germany) with the spectrum range of 400–4000 cm^−1^. The pHpzc of BMO was determined by Zetasizer Nano (ZEN3690, Malvern, England), measured using 0.01 M NaCl aqueous solutions in the pH range of 3.0–10.0. These pH values were fixed with 0.1 mol/L HCl and NaOH aqueous solution. The suspension was injected into the sample tank with a syringe, then the Zeta potential was measured in the sample tank, and the curve of Zeta potential changing with pH was drawn. When Zeta potential is 0, the corresponding pH is the point of zero charge of the material (pHpzc).

## 3. Results and Discussion

### 3.1. Effects of Environmental Factors on Bacterial Growth and Mn Removal

**Figure 2:**
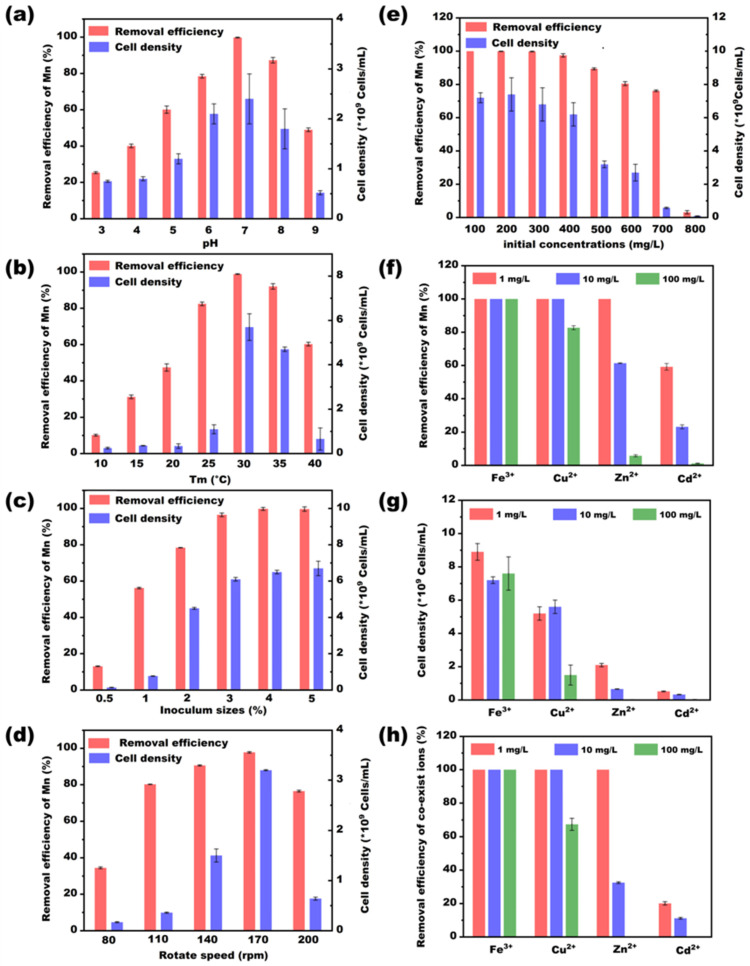
The influence of different environmental factors on bacterial growth and Mn removal. ((**a**): pH; (**b**): temperature; (**c**): inoculum size; (**d**): rotary speed; (**e**): initial Mn concentration; (**f**–**h**): different co-existing ions.)

The manganese-oxidizing bacterial consortium QBS-1 was enriched from the sediment in Qibaoshan mine. The different environmental factors that might affect the Mn(II)-oxidizing activity were investigated, and the results are shown in Figure 2. It is evident from Figure 2e that QBS-1 exhibited strong survival capabilities, with cell density exceeding 10^9^ cells/mL and the removal rates remaining at 80.5% even when the initial Mn concentration exceeded 600 mg/L. It is known that manganese is an energy resource for the growth and metabolism of Mn-oxidizing bacteria. But excessive Mn is toxic to microorganisms because it damages the structures of enzymes and disturbs osmotic balance. Thus, in the previous studies, the isolated MnOB performed well at oxidizing Mn^2+^ when the Mn concentrations were between 50 and 100 mg/L [7,33], but the removal efficiency decreased sharply if the Mn concentration was higher than 274.7 mg/L [16]. The excellent Mn resistance of QBS-1 in this study might be ascribed to a more stable system of the bacterial consortium and the long-term acclimation in the mining area and domestication experiments.

Figure 2 also displays the variations in cell densities and Mn-removal abilities of QBS-1 in the different pH, temperatures (Tm), inoculum sizes, and rotary speeds. The results showed that QBS-1 survived well in a pH range of 4.0–8.0, temperature range of 25–35 °C, inoculum size ranging from 2.0 to 5.0%, and rotary speeds ranging from 140 to 170 rpm, in which the cell densities of bacteria reached 10^9^ cells/mL. Unlike the findings of previous reports, the QBS-1 has a wider pH tolerance range (4.0–8.0), which provides good potential for removing manganese steadily in a variable environment. And the result confirmed that specific enrichment sites (acid mining area) provide an opportunity for bacteria to obtain a good acid resistance [13]. Moreover, the influence of different coexisting ions (Fe^3+^, Cu^2+^, Zn^2+^, Cd^2+^) on Mn-removal efficiency were also investigated in this study, and the results are shown in Figure 2f–h. As shown in Figure 2g,h, the QBS-1 was obviously inhibited when the Zn^2+^ or Cd^2+^ coexisted in the solution, especially when the concentrations reached 100 mg/L, in which the cell densities were only 10^7^ cells/mL and the Mn-removal efficiencies were lower than 10%. This might be due to the toxic effects of Zn^2+^ and Cd^2+^ on the microorganism. This finding is similar to a previously reported result indicating that Zn^2+^ and Cd^2+^ almost completely inhibited the Mn(II) oxidation of the consortium [34]. On the other hand, Fe^3+^ and Cu^2+^ had a positive effect on the bacterial growth, in which the cell densities were higher than 5.0 × 10^9^ cells/mL. As reported in similar studies, Fe^3+^ and Cu^2+^ were important co-enzymes of manganese oxidase; an appropriate concentration of Fe^3+^ and Cu^2+^ could improve the manganese-oxidation capacity of bacteria [34]. Moreover, good removal performances for Fe^3+^ and Cu^2+^ were also observed, which are possibly due to the adsorption and co-precipitation by biogenic Mn oxides [35].

### 3.2. Optimization Conditions for Mn-Removal Efficiency

To obtain the optimal theoretical conditions for Mn-removal efficiency, RSM was applied to evaluate and optimize the culture condition of the bacterial consortium. As shown in Table S5, the *p*-value of the model was less than 0.05 and the F-value was 5024.03, which means that the quadratic model was significant and applicable. The lack of fit was another important predictor of model. And in this study, the probability value of lack of fit was 0.0516 (not significant), further indicating the meaningfulness of this model. Moreover, the R^2^ value (0.9998) was in accordance with the adjusted R^2^ value (0.9996), proving that the experiment had high accuracy and strong reliability (Table S6). The polynomial equation generated for Mn-removal efficiency is given as follows:

Mn-removal efficiency = +99.17 − 8.14 × A + 6.88 × B + 10.29 × C − 0.2 × AB + 9.18 × AC − 6.75 × BC − 44.82 × A2 − 29 × B2 − 16.17 × C2(2)

Note: A (pH); B (temperature); C (inoculum size).

**Figure 3:**
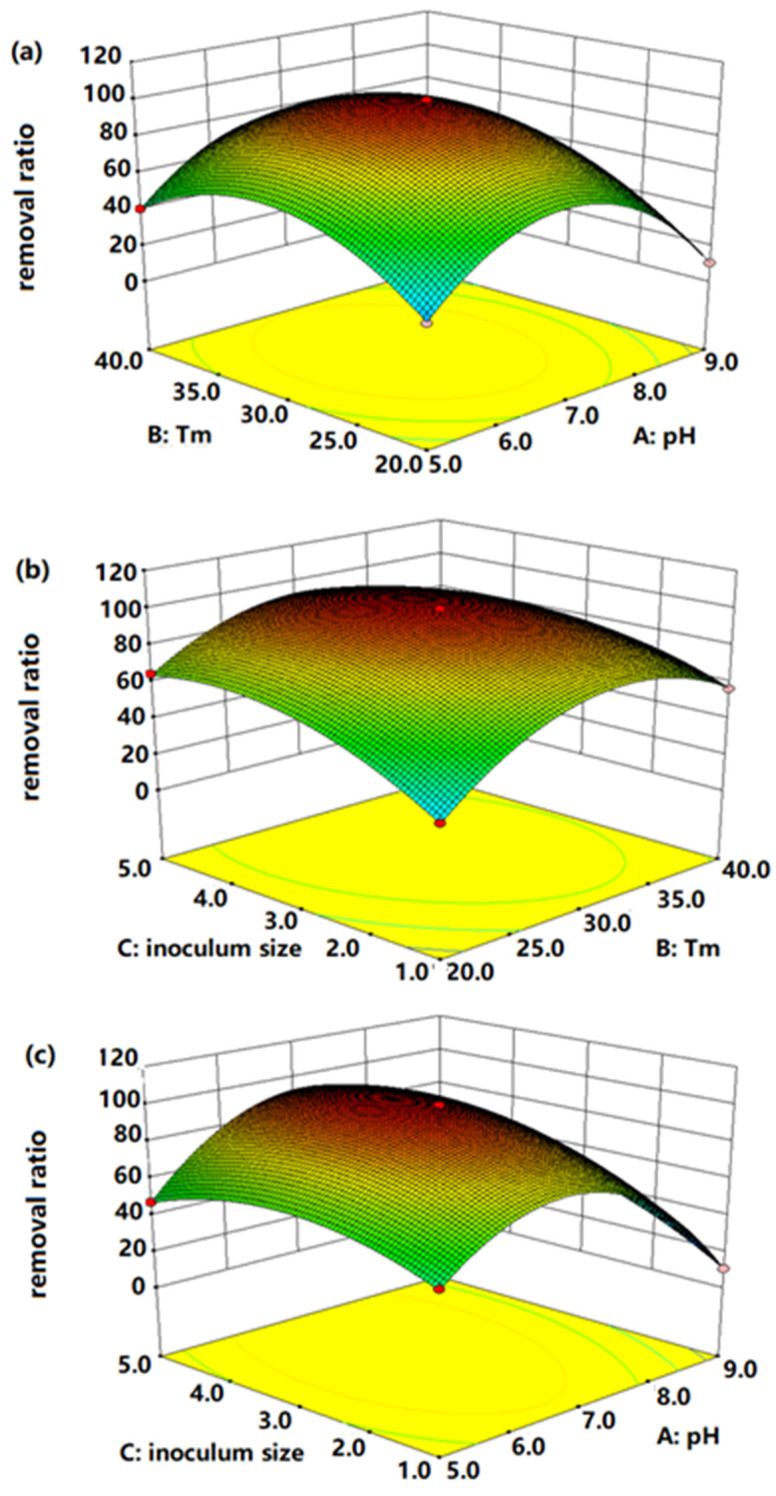
The 3D surface interaction of variable on the expression response using the Box–Behnken RSM design. ((**a**): pH; (**b**): Tm; (**c**): inoculum size.)

Three-dimensional surface plots and contour plots were used to study the interactive effects of three variables AB, AC, BC on Mn-removal efficiency. The elliptical contour plots in Figure 3 indicate a significant interaction between variables. As observed in Figure 3a, the removal efficiencies of Mn decreased obviously in the lower or higher levels. Good performances of bacteria were in the ranges of 30–35 °C and pH 6–8. Figure 3b depicts the interactive effects of Tm and inoculum size on the Mn removal. The results demonstrated that the bacterial consortium could maintain excellent Mn removal abilities when the Tm was 25–35 °C and inoculum size was 3–4%. Similarly, Figure 3c shows a significant interaction between pH and inoculum size, and the Mn removal rates were inhibited at their higher and lower levels. The steepest radian of Figure 3c confirms that the combination of pH and inoculum size had the most significant effects on the Mn removal. The most important issue of this part of the experiment was to establish the optimal culture conditions for Mn removal. According to the RSM analysis, it was determined that the best combination was a temperature of 29.38 °C, pH of 7.09 and inoculum size of 3.84%. Furthermore, the verification experiments were proposed under the optimal conditions, and the actual result (99.86%) was consistent with the predicted value (100%). Thus, the model of this study was credible and accurate.

**Figure 4:**
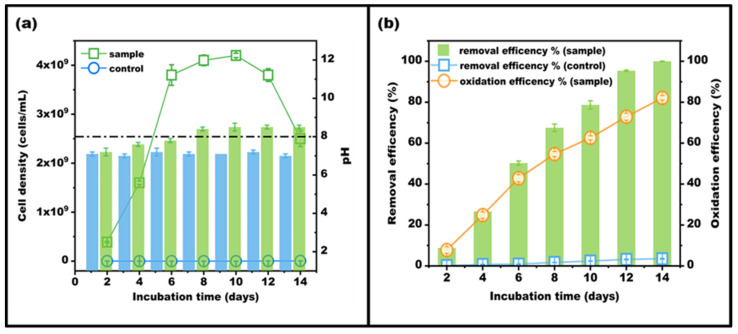
Changes in chemical factors in the culture ((**a**): Changes in cell density and pH; (**b**): Removal and oxidation efficiencies of Mn).

After optimizing the performance of the consortium, this study further investigated the dynamic changes in the chemical factors of incubation, and the results are shown in Figure 4. The cell densities of bacteria increased exponentially in the first 6 days, and the bacterial population was stable with a higher count (3.8–4.2 × 10^9^ cells/mL) during the plateau stage (6–12 days). Thereafter, a slight decline was observed, with a cell density of 2.5 × 10^9^ cells/mL on the 14th day. Similar trends appeared in the Mn removal and oxidation efficiencies. During the plateau stage, the removal efficiency of Mn increased from 50.27 to 95.42%, and the oxidation efficiency increased to 72.91%. The maximum removal efficiency of Mn was 99.99%, whereas there was no significant Mn removal in the control group (3.50%, after 14 days). Our results further demonstrated that bacterial consortium QBS-1 has a good ability for Mn remediation. However, although the Mn-removal efficiency was almost 100%, the oxidation rate was only 82.12%, indicating that other removal mechanisms participated in the Mn-removal process. Consistent with this, extensive previous studies have suggested that the adsorption on Mn oxides and the indirect oxidation induced by increased pH or dissolved oxygen could also promote the Mn elimination [7,26]. For example, *Lysinibacillus* sp. MK-1, a well-studied Mn-oxidizing bacterium, has superior Mn^2+^ removal ability (94.67%), and the metabolic mechanism was proved to have a comprehensive effect on bio-oxidation and adsorption [18]. Similarly, *Brachybacterium* sp. Mn32 firstly oxidized Mn(Ⅱ) to create Mn(Ⅲ) intermediates, and then adsorbed more Mn(Ⅱ) from the solution by the biogenic Mn oxides generated around the cell surfaces [36].

Besides that, another observed phenomenon was that the pH of cultures increased obviously from 7.2 (2nd day) to 8.5 (14th day). Similarly, Barboza et al. and Hullo et al. pointed out that the Mn-oxidizing bacteria *Stenotrophomonas* and *Bacillus subtilis* could induce an increased pH of medium through ammonification and further improve Mn oxidation [23,37]. Other studies further demonstrated that the increased pH might be ascribed to the consumption of CO_2_ and acid or the production of chemical oxidants (H_2_O_2_ or •OH) [13,26]. Hence, the increased pH favors the bacterial growth and the oxidation of Mn (II) in this study.

### 3.3. Dynamic Changes in Microbial Community

The high-throughput sequencing results were employed to evaluate the changes in microbial community diversity. The rarefaction curves of all samples reached saturation, indicating that the clonal libraries were sufficient to reflect the bacterial consortia (Figure S1). The analysis results demonstrated that both microbial community structures and compositions had a large discrepancy among different treatment periods (*p* < 0.05, *t*-test). In this study, principal coordinates analysis (PCoA) was used to compare the structures of the microbial community, in which the samples gathered together on the PCoA map if they had similar structures. As shown in Supplementary Materials (Figure S2), the samples at different stages were far apart from each other, suggesting a succession of bacterial communities during the treatment.

**Figure 5:**
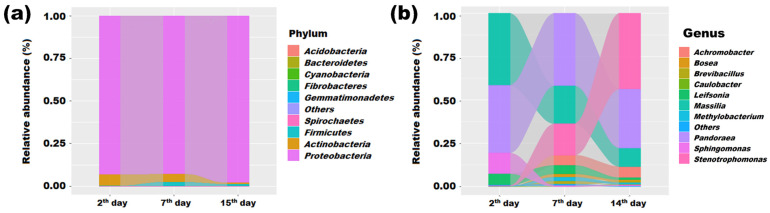
Relative abundance at phylum and genus level. ((**a**). relative abundance at phylum level; (**b**). relative abundance at genus level).

To further discover the potential players involved in manganese removal, the dynamic changes in the compositions of bacterial communities were investigated at phylum and genus level, and the results are shown in Figure 5a. The microbial consortia maintained a relatively stable state at the phylum level throughout the incubation process. *Proteobacteria*, *Actinobacteria* and *Firmicutes* were the most abundant phyla, accounting for over 99.9% of the total reads in each library. This finding is consistent with previous studies that have identified *Proteobacteria*, *Firmicutes* and *Actinobacteria* as the main Mn(II)-oxidizing bacteria [4,38]. Interestingly, the relative abundance of *Firmicutes* significantly increased after 2 days, from 0.16% (2nd day) to 2.17% (7th day) and 1.02% (14th day). *Firmicutes* are known to have superior heavy metal resistance due to their thick cell wall and the persistence of their endospores under stressful conditions [39,40]. For example, *Exiguobacterium* and *Anoxybacillus* have been implicated in manganese (II) oxidation and arsenic reduction [41,42], respectively. Therefore, the dominance and increasing trend of these bacteria suggest their potential role in manganese cycling metabolic processes and the enhancement of manganese-removal efficiency from contaminated water.

**Figure 6:**
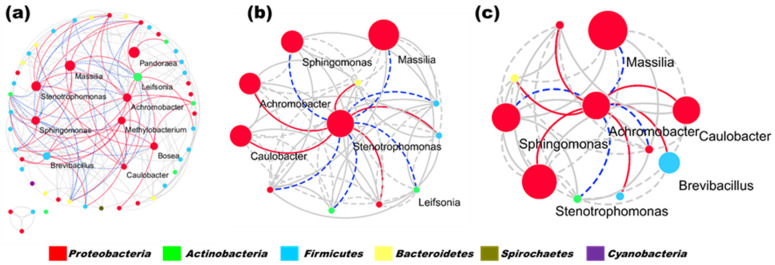
Co-occurrence network based on correlation analysis ((**a**): Profile clustering network of the 100 most abundant genera; (**b**): sub-networks of *Stenotrophomonas*; (**c**): sub-networks of *Achromobacter*. Circle size was proportional to the average abundance of the OUT. Red solid lines indicate positive relationships, while blue dotted lines indicate negative relationships. The different colors of nodes represent different phyla. Only genus names with a relative abundance in the top 10 are shown in the network).

Interestingly, the compositions of bacterial communities changed obviously at the genus level, suggesting that a succession of bacterial communities was unavoidable during the treatment. In bacterial consortia, many bacterial species possess the same biological functions. When destroyed by environmental changes, some bacteria may be lost or die while others who have the same functional genes are able to complement the loss of functions [43]. Hence, it was important to identify the increased bacteria which might be responsible for Mn elimination. As shown in Figure 5b, *Stenotrophomonas* and *Achromobacter* enriched obviously after 2 days. The relative abundance of *Stenotrophomonas* increased from 0.18% (2nd day) to 18.15% (7th day) and became the most abundance genus at the 14th day (43.59%). The genus *Achromobacter* was enriched from 0.05% (2nd day) to 5.94% (7th day) and 6.45% (14th day). Moreover, a network analysis was provided to analyze the various types of interactions between microorganisms. Figure 6a shows the connections among the 100 most abundant genera. There were 61 nodes with at least one connection in the molecular ecology networks. A total of 201 edges were identified, comprising 151 (75.12%) positive and 50 (24.88%) negative interactions. It was clear that the reactor microorganisms live together within complicated networks through various types of interactions, especially the bacteria among top 10 abundance, which comprised 37.31% of the edges of the whole network. In addition, due to the increasing abundance of *Stenotrophomonas* and *Achromobacter*, the interactions between these genera and other bacterial groups were also investigated (Figure 6b,c). Concordantly, the number of edges linked with *Stenotrophomonas* and *Achromobacter* were 12 and 8, respectively, which was more than other bacteria. A higher number of edges of bacteria means more important functions for the structural association of the bacterial community. Many strains associated with *Stenotrophomonas* and *Achromobacter* were reported to be able to oxidize Mn ions to create manganese oxides (Mn^3+^/Mn^4+^) [23,44]. For instance, Barboza et al. had screened manganese-oxidizing bacteria from a manganese mine and five of them belonged to *Stenotrophomonas*. One of the isolates, *Stenotrophomonas* sp. 7P, demonstrated high efficacy in removing Mn^2+^ from the medium, with a removal rate of 70.9% [23]. Similarly, *Achromobacter* sp. ty3-4 was a MnOB which could demonstrably oxidize 20 mM Mn^2+^ to produce manganese oxides within 80 h [45]. *Achromobacter* strain A14 was another reported MnOB with a Mn^2+^ oxidation rate of 0.373 mg·L^−1^·h^−1^ [44]. Besides this, the members of *Brevibacillus*, *Bosea* and *Caulobacter* were also demonstrated to have Mn-oxidizing capacities, and their relative abundances were increased obviously during the whole treatment [46,47,48]. Therefore, the enrichment of these Mn-oxidizing bacteria suggested that they may play vital roles in Mn removal. However, when compared with previously reported Mn-oxidizing bacterial consortia, the dominant genus of QBS-1 was different from previous studies [34,35]. This might be ascribed to the distinct screening sources for bacterial consortia, in which the mine area endowed QBS-1 with a higher Mn resistance and removal capability.

### 3.4. Possible Removal Mechanisms

After being cultured for 7 days, the suspensions of the bacterial consortium were prepared for morphology analysis using SEM. Figure S3a shows that bacteria form no aggregates on the surface of cells when cultured without Mn ions. However, Figure S3b shows clearly that some particulate matters were encrusted on the outer surface of cells when the bacterial consortium was cultured with Mn^2+^ ions. Consistent with this phenomenon, a mass of brown solid was obviously precipitated at the bottom of the flask after QBS-1 was cultured for 14 days. It has been reported that both the particles on the bacteria surface and the sediments at the bottom were biogenic Mn oxides, which were produced by Mn-oxidizing bacteria [49]. To further identify the character and probable functions of these biogenic Mn oxides (BMO), a series of analysis methods were carried out in this study.

**Figure 7:**
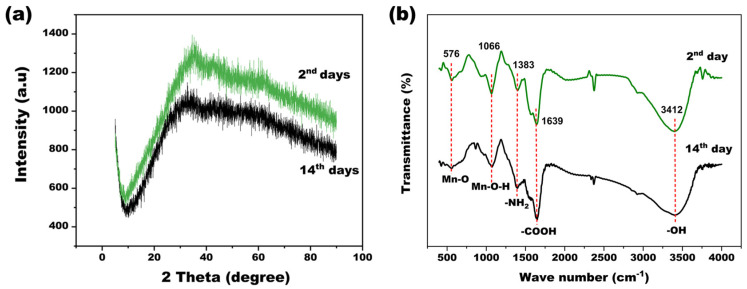
XRD, FTIR spectra of sediment on the 2nd day and 14th day. ((**a**): XRD analysis; (**b**): FTIR analysis).

Firstly, SEM-EDS and TEM were used to characterize the external and internal morphologies of BMO (Figures S4 and S5). According to the SEM image, the BMO sediments exhibited uniformly nanoparticles on the second day, and turned into agglomerates and massive structures after being cultured for 14 days, which resulted in a rough surface and porous structure. The EDS spectrum and Mn mapping further confirmed that the content of Mn element increased obviously from 0.82% (2nd day) to 16.85% (14th day). Moreover, the content of C, O and N indicated that the precipitate of culture may not be pure Mn oxides, and there was also some organic matter such as bodily bacteria or extracellular secretion in the sediments [14]. Furthermore, the TEM images (Figure S5) revealed that the clear lattice fringes exist in the manganese oxides, which display dark centers and bright edges. Consistent with our results, previous studies also demonstrated that the biogenic manganese oxides had an obvious lattice structure and this special structure was beneficial for its adsorbing effect [50]. However, XRD patterns (Figure 7a) showed that no typical crystalline form was detected in the BMO. The biological Mn oxides were primarily in amorphous nano-particulates [7], which might be because the crystalline formed in the BMO was too weak to be identified. The amorphous characteristic presented a higher specific surface area, which will further provide a richer redox-active center and larger ion accessible surface area [11,51]. In this study, the surface specific area of BMO was 103.09 m^2^/g, which was larger than previously reported BMO (marine microbial consortium, 41 m^2^/g; *pseudomonas putida* MnB1, 98 m^2^/g; and *Marinobacter* sp. MnI7-9, 0.47 m^2^/g) [48,52,53].

As discussed above, biogenic BMO was amorphous and had a larger specific surface, which might be an excellent sorbent and could eliminate greater quantities of dissolved Mn^2+^. Further studies were carried out to investigate the interaction between the BMO and metal ions by FTIR, XPS and pH_pzc_. As shown in Figure 7b, the characteristic absorption peaks of the two representative sediments all appeared at 576 and 1066 cm^−1^ for the Mn-O vibrations and Mn-OH bond, respectively. Moreover, the strong peaks at 1396, 1639 and 3412 cm^−1^ were assigned to -NH_2_, -COOH and O-H stretching vibrations of the free water, respectively [54,55]. The absorption peak of Mn-O and Mn-OH indicates that Mn atoms in BMO might have interacted with O via a coordination bond [26]. Function groups -NH_2_, carboxyl and hydroxyl can exchange or form complex bonds with heavy-metal ions in the fluid, and they enhanced the adsorption of contaminants. Specifically, the adsorption bands of -NH_2_, -COOH and O-H groups were shifted after 14 days, further implying that these function groups participated in Mn removal [49].

**Figure 8:**
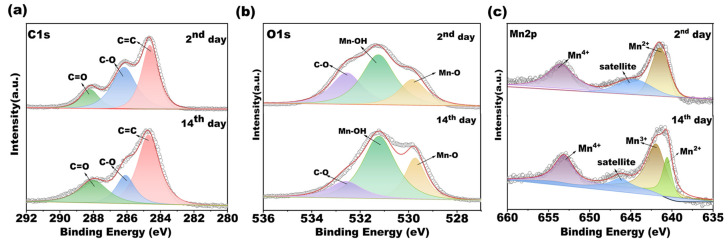
XPS spectra of sediment on the 2nd day (top) and 14th day (bottom). ((**a**): C 1s, (**b**): O 1s, (**c**): Mn 2p).

In addition, with the objective of analyzing the chemical states of elements on the manganese oxide layer generated by bacterial metabolism, XPS measurements on BMOs were taken. As shown in Figure 8a–c, the peaks corresponding to C 1s, O 1s and Mn 2p in BMO are clearly identified. The high-resolution C 1s spectrum was divided into three peaks at 284.5 (C=C), 286.4 (C-O) and 287.8 (C=O) [56,57]. Visibly, neither for the 2nd sample nor for the 14th sample, the C 1s spectra were mainly composed of the C=C peak. The O 1s XPS spectra depicts three fitting peaks (Figure 8b), which belong to the Mn-O, Mn-OH and C-O with binding energies of 529.6, 531.1 and 532.6, respectively [57]. Confirmed with our FTIR results, the data of XPS further demonstrated that the functional groups of Mn-O, Mn-OH, C-O and C=O existed on the surface of sediments, which might have a vital role in Mn removal. Figure 8c shows the XPS spectra for the binding energy of Mn 2p states, the Mn 2p was split by the spin–orbit interactions into the Mn 2p_3/2_ and Mn 2p_1/2_ peaks. When sampled on the 2nd day, the peak positions of Mn 2p for Mn^4+^ species was located at 653 eV, and the peaks at about 641.4 eV corresponded to the Mn^2+^ species followed with a satellite peak (645.4 eV) [58]. For the 14th-day samples, a major peak of Mn 2p_1/2_ was also observed at 653 eV (Mn^4+^), but the center of the Mn 2p_3/2_ peak was well fitted by two peaks at 640.7 and 641.9, respectively. The peaks at 640.7 corresponded to the chemical state Mn^2+^, while 641.9 belonged to Mn^3+^ species. To identify the content of elements on the samples, the relative amount of element species was calculated (Table S7). The surface concentration of Mn^2+^ decreased from 44.69 to 20.70%, whereas the content of high valent Mn (Mn^3+^/Mn^4+^) increased from 55.31 to 79.30% after 14 days. The results demonstrated that multiple-valence Mn species co-exist on the surface of BMO, which further confirmed our speculation that the removal of Mn was due not only to the function of bacteria oxidizing but also to the adsorption or co-precipitation. It has been demonstrated that the co-existence of multiple-valence Mn species was favored for the oxidation reduction reaction, resulting in the enhancement of Mn removal [59]. Moreover, the fact that the content of high-valent Mn increased obviously after being cultured for 14 days proved that the removal efficiency of Mn element was primarily ascribed to the oxidation of Mn (Ⅱ).

More useful information about the interaction between Mn ions and solid Mn oxides can be provided by the zeta potential analysis (Figure S6). The biogenic Mn oxide was found to have an isoelectric point of about 3.5. At lower pH (pH < PZC) values, the surface charge of the BMO and heavy-metal ions were both positive, suggesting that a purely repulsive coulombic interaction was experienced between the BMO and metal ions. By contrast, the surface of BMO was negatively charged at pH > 3.5, which was favorable for nonspecific adsorption of Mn ions through electrostatic attraction [60]. In this study, the pH of culturing situation was always higher than 3.5, implying that the intermolecular electrostatic attraction participated in the Mn-removal process.

### 3.5. Performance of Bacterial Consortium in the Real Wastewater

**Figure 9:**
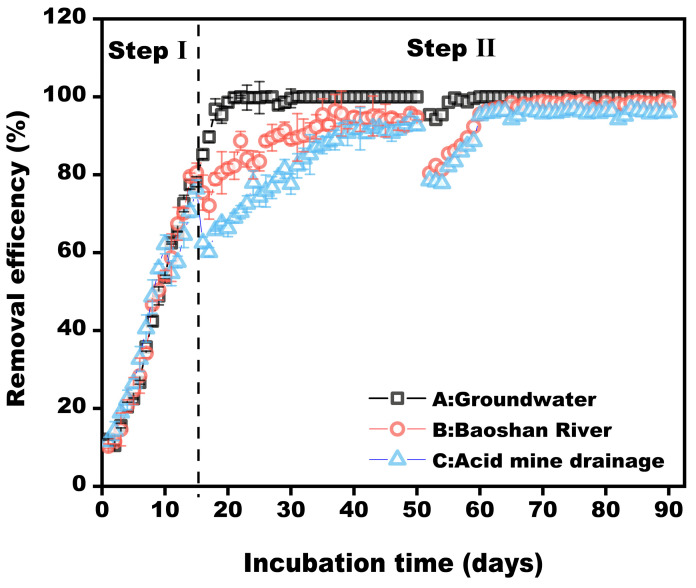
Removal efficiency of Mn in different types of wastewater by biofilter.

To confirm the performance of the bacterial consortium, three different levels of Mn contaminant wastewater were examined, and the results are presented in Figure 9. The three samples were simulated groundwater (4.54 mg/L, pH 7.4), Baoshan river (22.41 mg/L, pH 6.8) and acid-mine drainage (125.93 mg/L, pH 4.4). For stage Ⅰ (0–15), the acclimated bacterial consortium was used as the initial inoculum, and the synthetic Mn-contaminated wastewater was used as seeding medium to shorten the start time of the biofilter. After 15 days, the Mn removal ratios reached approximately 80% for all reactors, and no significant discrepancy appeared during this period. This indicated that all biofilters were started and the bacterial consortium QBS-1 was successfully immobilized in the biofilters. For stage II (16–50), after the real wastewater was pumped into the biofilters, Mn-removal efficiencies were decreased slightly in filter B (75.4%) and C (62.5%). These results are consistent with previous reports of decreasing Mn removal by real wastewater. This decrease in removal could be attributed to lower pH or higher Mn(II) concentration, which may suppress the activity of bacteria [4,27]. Nevertheless, the mature biofilter could recover its excellent performance after a period of adaptation. As shown in Figure 9, the removal ratios of Mn were greater than 90% in biofilter B and C when operated for 32 days and 38 days, respectively. This might be because the MnOB consortium gave the biofilter a more complex ecosystem, as in the results of the network analysis [34]. In a mature biofilter, the ecosystem function is usually redundant, which means different bacterial species possess the same biological functions. And even if some of them die or are lost due to the changing inflow, the fact that the others possess the same functions would complement the loss of functions immediately. This is why the MnOB biofilter could recover its removal capacity rapidly in this study. Hence, the continuous experiments also demonstrate that the bacterial consortium QBS-1 is a good inoculum and has the potential to remove Mn contamination steadily and effectively.

## 4. Conclusions

This work demonstrated that the bacterial consortium QBS-1 has excellent resistance to acid, which could eliminate Mn efficiently in a pH range from 4.0 to 8.0. *Stenotrophomonas* and *Achromobacter* are core genera of QBS-1 and play indispensable roles in Mn removal. Moreover, the probable Mn removal mechanism was the combined effects of bacteria oxidation, BMO adsorption, sediment co-precipitation and electrostatic binding, in which the bacteria oxidation was considered to play a dominant function. This study provided a novel method to enhance performance of Mn removal by MnOB and highlights the potential application of this bacterial consortium in environmental remediation, even in acid mining areas.

## Data Availability

Data are unavailable due to privacy.

## Supplementary Materials

The following supporting information can be downloaded at https://www.mdpi.com/article/10.3390/microorganisms11092185/s1, Table S1: Experimental range of the three variables studied using box-behnken design in terms of actual and coded factors; Table S2: RSM design table; Table S3: Chemical composition of Mn-contaminated wastewater; Table S4: Composition and preparation of the synthetic Mn-contaminated wastewater; Table S5: ANOVA statistics for the fitted quadratic polynomial of light intensity (Partial sum of squares-Type III); Table S6: Fit Statistics; Table S7: The relative amounts of element species (%) on the surface of sediments; Figure S1: Rarefaction curves of species numbers for every group; Figure S2: Principal co-ordinates analysis (PCoA) of bacterial community structure at different treatment stages; Figure S3: SEM images of bacterial consortium at different culturing conditions ((a). cultured 7 days without Mn(II) (b). cultured 7 days with 500 mg/L Mn(II)); Figure S4: SEM, EDS spectrum, and Mn-mapping images of sediments at 2nd day and 14th day ((a) and (b) were SEM images of sediments cultured 2nd and 14th day; (c) and (d) were EDS spectrum images of sediments cultured 2nd and 14th day; (e) and (f) were Mn-mapping images of sediments cultured 2nd and 14th day); Figure S5: TEM images of sediments at 14th day; Figure S6 Zeta potential of BMO at different pH values. References [26,61,62] are cited in the supplementary materials.
