# Supplementary material for: Enhancing the Mn-Removal Efficiency of Acid-Mine Bacterial Consortium: Performance Optimization and Mechanism Study
Source: Microorganisms. 2023 Aug 30;11(9):2185. doi: 10.3390/microorganisms11092185 (PMC10535970; doi:10.3390/microorganisms11092185)
Supplement: Supplementary file 1 [file microorganisms-11-02185-s001.zip › microorganisms-2564054-supplementary.pdf]

Supported Information

Enhancing the Mn-Removal Efficiency of Acid-Mine Bacterial Consortium: Performance Optimization and Mechanism Study

Dongmei Hou*, LanZhang, Chuncheng Li, Lutong Chen and Jianping Zou *

National-Local Joint Engineering Research Center of Heavy Metals Pollutants Control and Resource Utilization, Nanchang Hangkong University, Nanchang 330063, China;

zl15103424308@163.com (L.Z.); 15847761908@163.com (C.L.);

1780679929@126.com (L.C.)

* Correspondence: hou_dong_mei@126.com(D.H); zjp_112@126.com(J.Z.)

1. Acclimation of Mn(II) oxidizing consortium

The bacterial culture (2 mL) was transferred into PYCM medium with different concentrations of Mn (II), which were increased as 100, 200, 300, 400, 500, 600 in sequence. When the Mn (II) concentration was 400 mg/L or higher, the mixed bacterial culture was also acclimated repeatedly at the same concentration of Mn (II). When the the bacteria density reached to 1 $\times 10^9$ cells/mL at 600 mg/L Mn (II), the acclimation process finish.

2. DNA extraction, amplification, sequencing and analysis

The bacteria were harvested from 50 mL samples (including cultured medium and sediment) by centrifuged (*10000 ×g*) for 10 min. Total genome DNA was extracted using CTAB/SDS method. After determinate the concentration by 1% agarose gel, DNA samples were diluted to 1ng/μL using sterile water.

The V3-V4 region of bacterial 16S rRNA gene was amplified by PCR using the primers 314F (5'-CCTAYGGGRBGCASCAG-3') and 806R (5'-GGACTACNNGGGTATCTAAT-3'). Purified PCR amplicons were carried out with Illumina HiSeq platform [1]. Raw fastq files were optimized by following steps: remove sequences with low quality (with a length <220bp), with ambiguous base "N", and score lower than 20. After that, high quality sequences with ≥ 97% similarity were clustered into the same operational taxonomic units (OTUs) for further annotation [2]. The abundance table was exported to the R environment, and the statisticalan alysis and visualization was performed with the phyloseq, ggalluvial, ggplot2 and reshape2 libraries. The principal co-ordinates analysis (PCoA) using bray_curtis distance was

plotted to compare the microbial community structure during treatment. Only those genera which with relative abundance in the top 100 were taken into consideration in co- occurrence networks, which were based Spearman's rank correlations in R environment with the psych package. The networks were processed with the Cytoscape software, and described as the previous study [3].

Table S1. Experimental range of the three variables studied using box-behnken design in terms of actual and coded factors.

Component	Level		
	-1	0	+1
pH	5	7	9
Tm (°C)	20	30	40
inoculum size (%)	1	3	5

Table S2. RSM design table.

Run	Coded variables			Responses
	pH	Tm	inoculum size	removal efficiency (%)
1	-1	1	0	40.7
2	-1	0	-1	45.7
3	-1	-1	0	25.6
4	0	0	0	99.6
5	0	-1	1	64.8
6	-1	0	1	47.5
7	1	-1	0	10.4
8	1	0	1	49
9	0	0	0	99.2
10	0	0	0	98.9
11	0	0	0	99.6
12	1	0	-1	10.5
13	0	0	0	99.1
14	0	0	0	98.6
15	0	1	-1	56.7
16	0	-1	-1	30.3
17	0	1	1	64.2
18	1	1	0	24.6

Table S3. Chemical composition of Mn-contaminated wastewater.

Samples	description	pH	Mn (mg/L)	Cd (mg/L)	Zn (mg/L)	Cu (mg/L)
Sample 1	Simulated groundwater	7.4	4.54±1.20	0.02±0.01	0.83±0.10	0.47±0.15
Sample 2	upstream water of Baoshan River	6.8	22.41±1.60	0.9±0.02	7.55±1.21	1.35±0.26
Sample 3	Acid mine drainage in Qibaoshan Mine	4.4	125.93±5.8	8.21±1.40	58.89±3.63	36.27±2.32

* Simulated groundwater was a mixture of tap water and $MnSO_4 \cdot H_2O$.

Table S4. Composition and preparation of the synthetic Mn-contaminated wastewater.

Synthetic AMD medium	
Modified PYCM medium	500 mL
Mn (II) solution: $MnSO_4 \cdot H_2O$	125 mg/L
Cd (II) solution: $CdSO_4 \cdot 8H_2O$	8 mg/L
Zn (II) solution:$ZnSO_4 \cdot 7H_2O$	60 mg/L
Cu (II) solution:$CuSO_4 \cdot 5H_2O$	36 mg/L
pH	4

1. The modified PYCM medium (exclude $MnSO_4 \cdot H_2O$ and Ammonium ferric citrate) was sterilized by autoclaving 20 min at 120°C.

2. The modified PYCM medium was supplemented with the metal solution, the final concentration of metal ions were Mn 125 mg/L, Cd 8 mg/L, Zn 60 mg/L, Cu 36 mg/L for each flask. The metal solution was filtered through 0.22μm filters to removal bacterial.

Table S5. ANOVA statistics for the fitted quadratic polynomial of light intensity (Partial sum of squares-Type III).

Source	Sum of squares	df	Mean square	F-value	p-value	
**Model**	18317.32	9	2035.26	5024.03	<0.0001	significant
A-pH	5460.13	1	529.75	1307.69	<0.0001	
B-Tm	378.125	1	378.125	933.40	<0.0001	
C-inoculum size	846.66	1	846.66	2089.98	0.5472	
AB	0.16	1	0.16	0.395	<0.0001	
AC	336.72	1	336.72	831.20	<0.0001	
BC	182.25	1	182.25	449.88	<0.0001	
A^2	8766.14	1	8766.14	21639.22	<0.0001	
B^2	3668.76	1	3668.76	9056.35	<0.0001	
C^2	1141.07	1	1141.073	2816.74	<0.0001	
**Residual**	3.2408	8	0.4051			
Lack of Fit	2.47	3	0.8225	5.32	0.0516	not significant
Pure Error	0.7733	5	0.1547			
**Cor Total**	18320.55	17				

Table S6. Fit Statistics.

Std.Dev	Mean	C.V.%	R^2	Adjusted R^2	Predicted R^2	Adeq Precision
0.6365	59.17	1.08	0.9998	0.9996	0.9978	186.8227

Table S7. The relative amounts of element species (%) on the surface of sediments.

Samples	C=C	C-O	C=O	Mn-OH	Mn-O	C-O	Mn^{2+}	Mn^{3+}	Mn^{4+}
2nd day	48.25	35.52	16.23	37.57	20.56	41.87	44.69	0	55.31
14th day	53.17	16.08	30.75	63.10	25.51	11.39	20.70	51.00	28.30

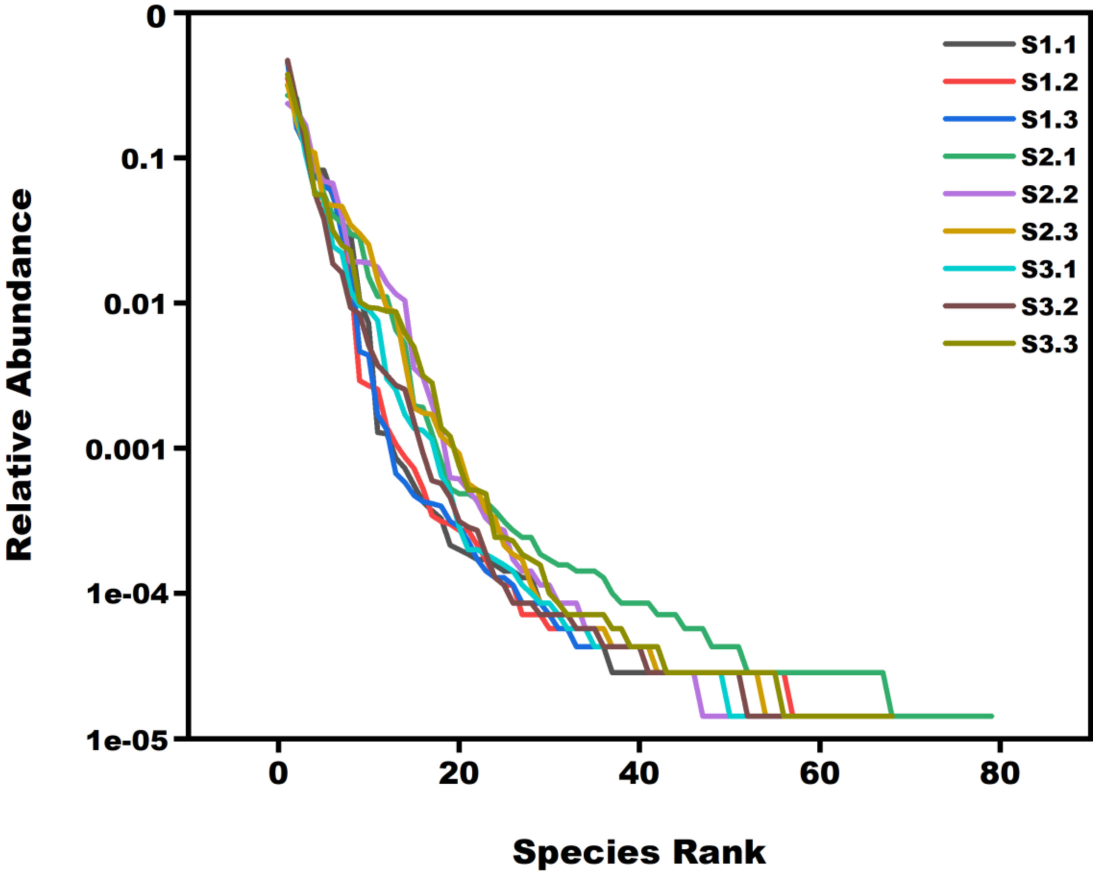

Figure S1. Rarefaction curves of species numbers for every group.

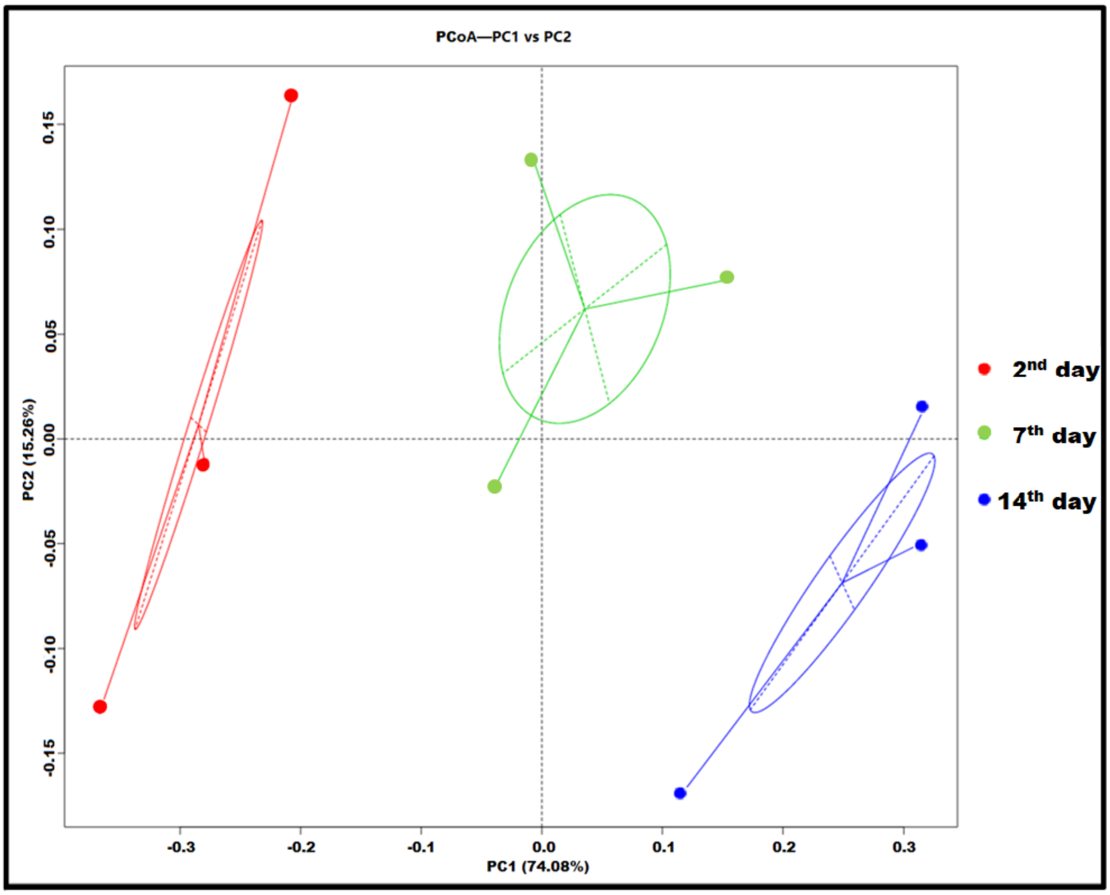

Figure S2. Principal co-ordinates analysis (PCoA) of bacterial community structure at different treatment stages.

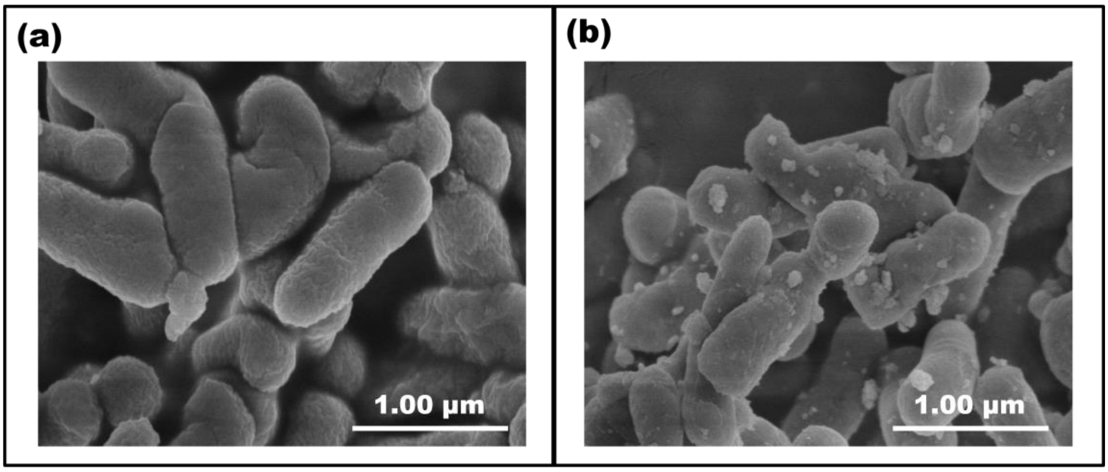

Figure S3. SEM images of bacterial consortium at different culturing conditions ((a). cultured 7 days without Mn(II) (b). cultured 7 days with 500 mg/L Mn(II)).

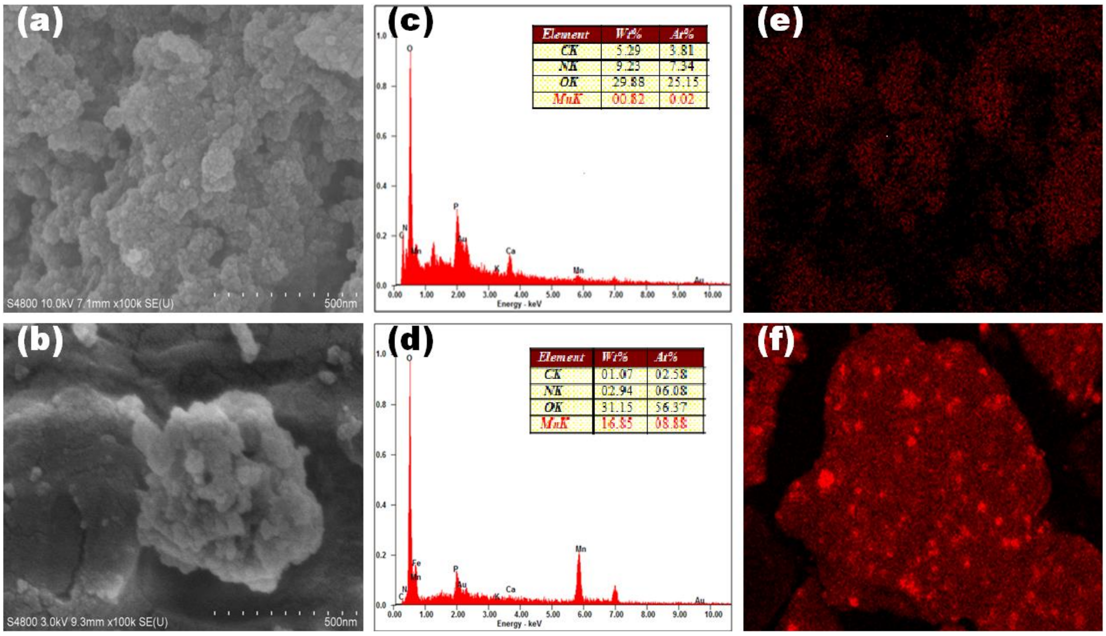

Figure S4. SEM, EDS spectrum, and Mn-mapping images of sediments at 2nd day and 14th day ((a) and (b) were SEM images of sediments cultured 2nd and 14th day; (c) and (d) were EDS spectrum images of sediments cultured 2nd and 14th day; (e) and (f) were Mn-mapping images of sediments cultured 2nd and 14th day.)

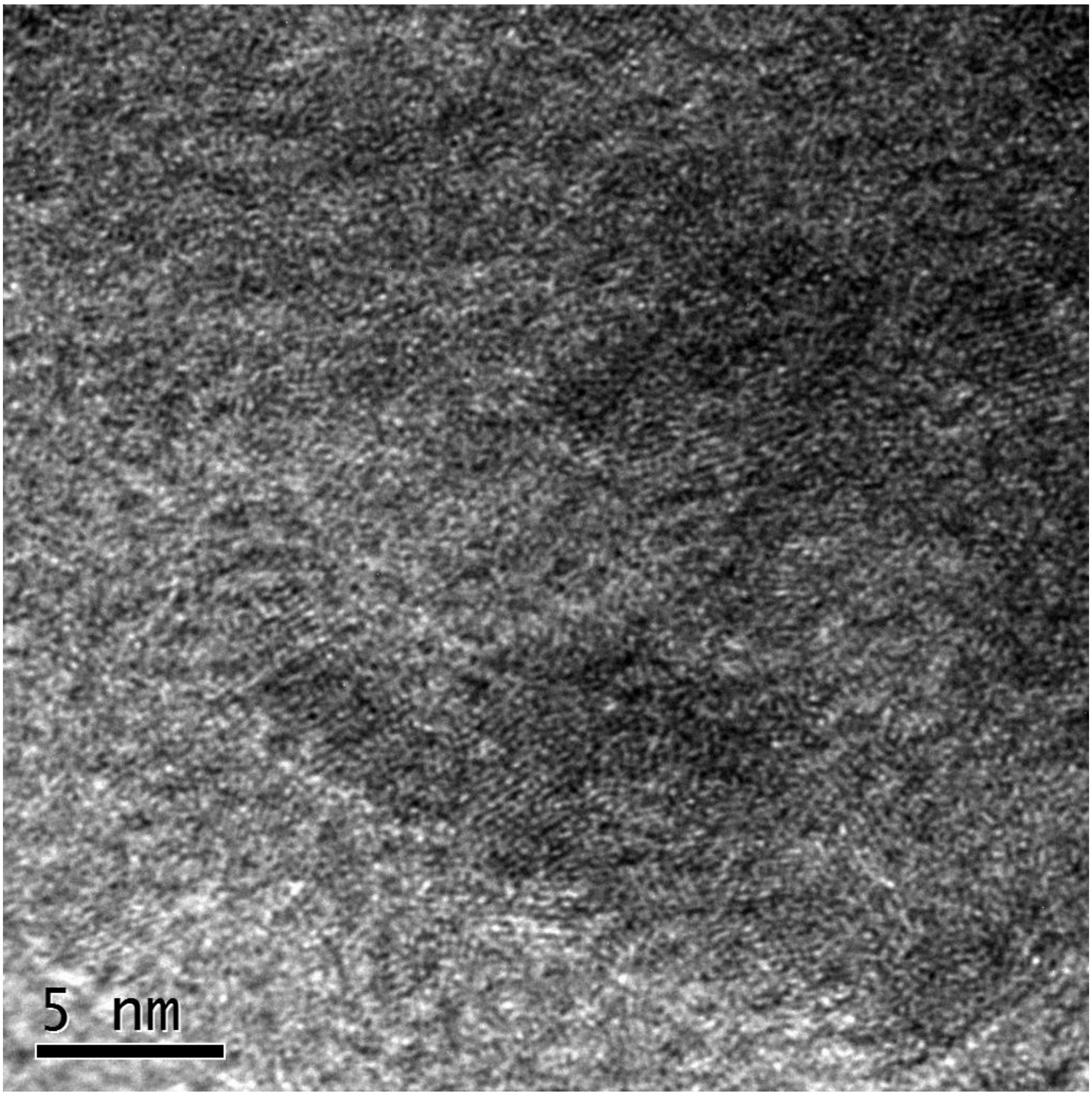

Figure S5. TEM images of sediments at 14th day.

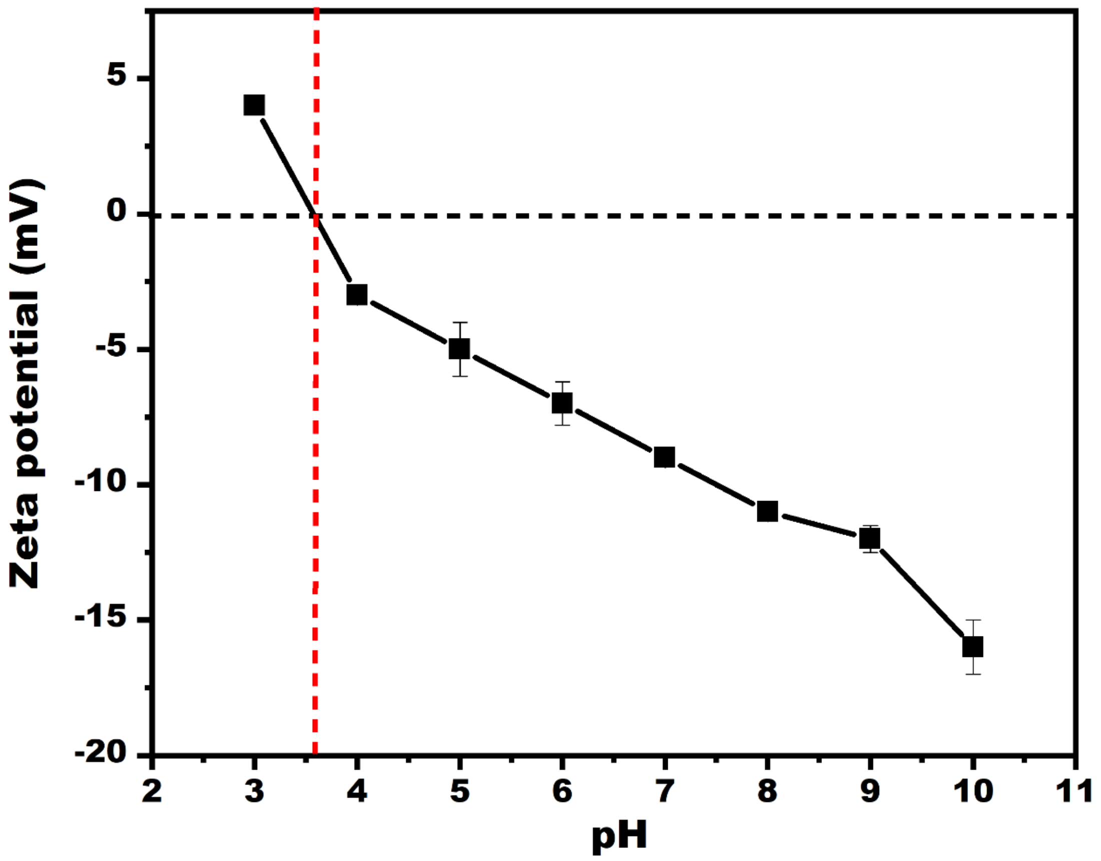

Figure S6. Zeta potential of BMO at different pH values.

References

[1] Hou D, Zhang P, Wei D, et al. Simultaneous removal of iron and manganese from acid mine drainage by acclimated bacteria[J]. Journal of Hazardous Materials, 2020, 396: 122631.

[2] Chen, H., Wang, Y., Chen, Z., et al. Effects of salinity on anoxic–oxic system performance, microbial community dynamics and co-occurrence network during treating wastewater. Chem. Eng. J. 2023, 461, 141969.

[3] Sun W, Xiao E, Kalin M, et al. Remediation of antimony-rich mine waters: Assessment of antimony removal and shifts in the microbial community of an onsite field-scale bioreactor[J]. Environ Pollut, 2016, 215: 213-222.
